# A perspective on human cell models for POLG-spectrum disorders: advantages and disadvantages of CRISPR-Cas-based vs. patient-derived iPSC models

**DOI:** 10.1515/medgen-2021-2090

**Published:** 2021-12-03

**Authors:** Cagla Cakmak, Hans Zempel

**Affiliations:** Institute of Human Genetics, Faculty of Medicine and University Hospital Cologne, University of Cologne, Cologne, Germany

**Keywords:** human cell model, CRISPR, iPSC, POLG, neurodegenerative disease, mitochondriopathy

## Abstract

Neurogenetic diseases represent a broad group of diseases with variable genetic causes and clinical manifestations. Among these, polymerase-gamma (POLG)-spectrum disorders are relatively frequent with an estimated disease frequency of ∼1:10.000. Also, mutations in the *POLG* gene are by far the most important cause for mitochondriopathy. POLG-spectrum disorders usually result in progressive loss of brain function and may involve severe and deadly encephalopathy, seizures, and neuromuscular disease, as well as cardiac and hepatic failure in some cases. Onset of disease may range from birth to late adulthood, and disease duration ranges from weeks in severe cases to decades. There is no curative treatment; current animal models do not faithfully recapitulate human disease, complicating preclinical therapeutic studies. Human-based preclinical model systems must be developed to understand the human disease mechanisms and develop therapeutic approaches. In this review, we provide an overview of the current approaches to model neurogenetic disorders in a human cellular and neuronal environment with a focus on POLG-spectrum disorders. We discuss the necessity of using neuronal cells and the advantages and pitfalls of currently available cell model approaches, namely (i) CRISPR-based (i. e., genetically engineered) and induced pluripotent stem cell (iPSC) (i. e., stem cell like)-derived neuronal models and (ii) the reprogramming of patient-derived cells into iPSCs and derived neurons. Despite the fact that cell models are by definition *in vitro* systems incapable of recapitulating all aspects of human disease, they are still the reasonable point of start to discover disease mechanisms and develop therapeutic approaches to treat neurogenetic diseases.

## Introduction

Generating cellular and mouse models for neurological diseases enables us to uncover disease mechanisms as well as develop therapeutic approaches also for neurogenetic diseases that are difficult to treat. Yet, neurological diseases are hard to model due to highly complex disease mechanisms and often age-dependent disease onset. Current animal and cell models are limited as they recapitulate only some aspects of human disease. Mitochondrial diseases are considered even more challenging to diagnose, treat, and model because their symptoms can vary tremendously even among patients with the exact same mutation [1]. Clinical variability can be due to mitochondrial heteroplasmy (meaning different amounts of mutated mitochondria/mitochondrial DNA in different tissues), the type of mutation (whole gene deletion vs. single-nucleotide exchange, size of repeat expansion, etc.), or whether gene mutations are homozygous or compound heterozygous for autosomal recessive disease. The latter is true, e. g., also for polymerase-gamma (POLG)-spectrum disorders (POLG-SDs), where compound heterozygosity of the two POLG mutations may result in a more severe phenotype/shorter lifespan than the same mutations when present homozygously [2]. Also, disease onset can range from infancy to the forties. POLG-SDs are the result of a defective POLG enzyme, the only mammalian mitochondrial DNA polymerase encoded by the gene *POLG* on chromosome 15 [3]. Therefore, expression of proteins encoded by the mitochondrial genome, maintenance of a robust oxidative phosphorylation, and other physiological functions are naturally related with POLG function. Bindoff et al. showed that POLG-induced complex I dysfunction leads to oxidative damage [4]. Considering the crucial dependence of neuronal cells on mitochondria, associated phenotypes are mainly neurological, although in severe forms, other organs (in particular the liver, heart, and muscles) can be heavily involved [1]. POLG-SDs caused by mutations in *POLG* are usually autosomal recessive and range from Alpers–Huttenlocher syndrome (AHS, fatal progression usually within weeks to a few years after birth), ataxia neuropathy spectrum (ANS), and myoclonic epilepsy myopathy sensory ataxia (MEMSA) to often late-onset progressive external ophthalmoplegia (PEO) [2], [3]. There is no effective treatment for POLG-SDs, and symptomatic treatments cannot prevent the fatal progression of the more severe forms of the disease. Mouse models do not recapitulate the human disease and pathomechanisms, and possible treatment approaches are under- or unstudied.

**Figure 1:**
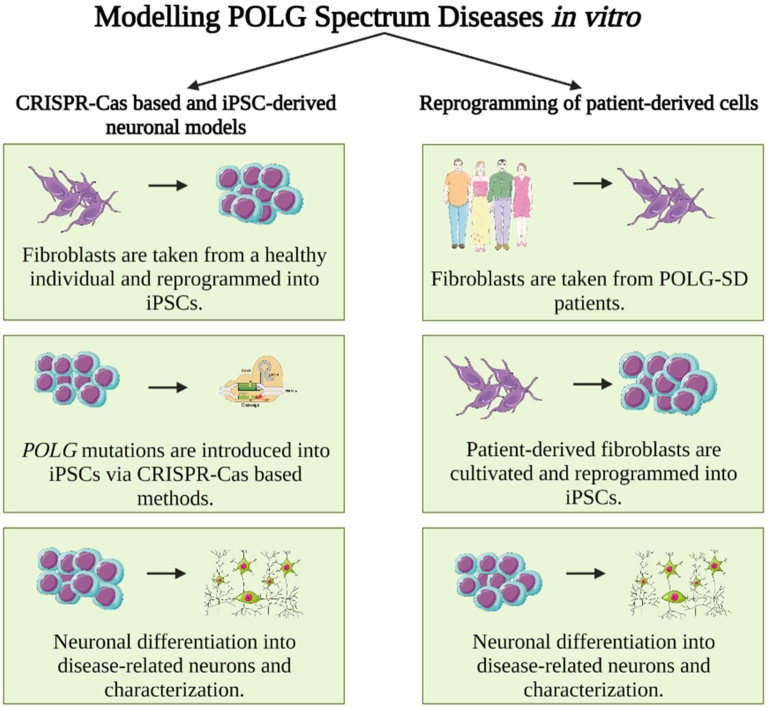
Schematic depiction of steps necessary for the study of human (iPSC-derived) neurons for the modeling of POLG-SDs in disease-relevant neurons. *Left*: Induced pluripotent stem cells (iPSCs) are obtained from either commercial suppliers or biobanks, which has the advantage that these cells are usually widely used and well characterized. However, disease-specific (i. e., here POLG) mutations have to be introduced via gene editing, e. g., through CRISPR-Cas technology. The original iPSC line can serve as isogenic control. *Right*: iPSCs can also be generated directly from affected patients by reprogramming of patient fibroblasts, which has the advantage that the obtained iPSC line is obtained from a patient whose genetic constitution in principle permits disease manifestation. Obtained cells should be thoroughly characterized to rule out reprogramming artifacts. Isogenic controls – if desired – have to be created via correcting the patient-specific mutation to obtain a corresponding (POLG) wild-type control.

Current approaches to model POLG-SDs in a human cellular and neuronal environment include (i) CRISPR-Cas-based and iPSC-derived neuronal models and (ii) reprogramming of patient-derived cells into neurons. CRISPR-Cas technologies allow a broad range of gene modifications and are applicable to proliferative cells, a. o. standard neuronal cell lines, but, importantly, also human induced pluripotent stem cells (iPSCs) [5]. This makes CRISPR-Cas-based gene editing a valuable tool for neurogenetic diseases, since iPSCs can be differentiated into neurons of various subtypes, including neurons primarily affected by POLG-SDs (e. g., cortical GABAergic neurons) [5], [6]. The other approach, reprogramming of patient-derived cells into neurons, has the advantage that (i) initially no genome engineering is required (but see below for gene editing of patient-derived iPSCs into isogenic wildtype controls) and (ii) one can be sure that the genetic makeup of the reprogrammed cell, in principle, permits disease development in humans [7]. Here, we briefly discuss the necessity of using neuronal cells and discuss advantages and pitfalls of patient-derived cells as well as genetically engineered cell lines/iPSCs (for a schematic depiction see also Figure 1).

## CRISPR-Cas-based and iPSC-derived neuronal models

POLG-SDs primarily affect neurons of the central nervous system. Neuroradiological findings revealed that abnormalities include thalamic, basal ganglia, and cerebellar lesions and generalized cortex and brain atrophy [8], [9]. Therefore, disease-related neuronal cells are the preferential choice in POLG-SD cell models. In recent years, improvements in the ability to reprogram patient-derived cells into iPSCs provided a novel method to develop disease-related cellular models [4], [10]. These stem cell-like cells are now commonly used in neuroscience, cardiology, disease modeling, and even drug discovery studies. iPSCs are able to proliferate indefinitely in vitro, providing an unlimited source of cells similar to stem cells. Regardless of the original tissue iPSCs were generated from, they can differentiate into any cell type of the human body [11]. Life scientists have developed detailed protocols to successfully differentiate iPSCs into neurons in the past years [12]. One of the greatest advantages of the iPSCs is that gene-editing technologies allow scientists to manipulate the genetic background of iPSCs to mimic, e. g., POLG-SDs by introducing disease-associated *POLG* mutations. These cumulative advances in iPSC technology have led to major accomplishments in uncovering disease mechanisms for other neurodegenerative diseases, e. g., Alzheimer’s and Huntington’s [12], [13].

CRISPR-Cas-based cellular modeling begins with the generation of iPSCs from somatic cells, e. g., fibroblasts taken from a healthy individual. These somatic cells are usually reprogrammed into iPSCs by introducing products of specific sets of pluripotency-associated genes, the so-called Yamanaka factors (*OCT4*, *SOX2*, *KLF4*, and *c-MYC*) [14], [15]. The use of non-integrative programming strategies via Sendai virus or episomal plasmids (a. o. also Adenovirus with extremely low reprogramming efficiency (∼0.001 %), cytoplasmic RNA, recombinant proteins and microRNAs) [16] are the most common and very efficent reprogramming strategies, while more dated integrative programming techniques (e. g. lentiviral or retroviral reprogramming) are still in use. All of the described methods have, however, limitations [17]. CRISPR-Cas-based genome editing enables scientists to modify genes/genomic loci by introducing a mutation, creating a deletion or any desired modification detected in patients. In particular, c.1399G>A (A467T) and c.2243G>C (W748S) mutations [18], [19], observed in most POLG-related patient cases, can be introduced into iPSCs. These genetically engineered iPSCs can further be differentiated into disease-relevant neuronal cells using specific differentiation protocols. These protocols mainly rely on sequential additions of neurotrophins like glial cell line-derived neurotrophic factor (GDNF) and brain-derived neurotrophic factor (BDNF) [20], while others also make use of exogenous expression of neuronal subtype-specific transcription factors that force differentiation into neurons of brain regions affected in POLG-SDs [12].

CRISPR-Cas based systems (like currently mainly used CRISPR/Cas9, and newer methods as Cas9 prime editing or CRISPR/Cas9-loxP mediated editing) have 40–80 % target efficiencies when recent approaches such as electroporation are applied. However, the challenge arises from differences in transfection or electroporation rates, DNA repair fidelity, variable promoter and exonuclease activity [21]. In addition, the application of the CRISPR/Cas9 editing procedure might be time and effort consuming depending on the cell line and the genetic modification. Genetically engineered cells require genetic confirmation of the successful introduction of the desired mutation by PCR and sequencing, the absence of off-target effects and genetic aberrations, and subsequent biochemical confirmation of differentiated cells with Western blotting (WB) and/or immunofluorescence (IF) microscopy analysis of neuronal marker proteins. Another main problem of CRISPR-Cas-based methods to model POLG-SDs is the heterogeneity of the genetic composition of the patients and variable clinical manifestations of the disorders. It has been commonly observed that patients do not display the same phenotype even though they carry the same mutation(s). This is also true vice versa; some patients share the same clinical manifestations but carry diverse *POLG* mutations [2], [22]. Patients suffering from AHS, one of the most severe POLG-SDs, develop recurrent seizures (intractable epilepsy), psychomotor regression, and liver disease [2], [3]. AHS is associated with homozygous or compound heterozygous mutations [3], [23] in *POLG*. However, for the two most common mutations, A467T and W748S, age of death is relatively high, with an average of 20 to 25 years [23]. Currently there is no convenient or disease-relevant cellular model for AHS. In addition, compound heterozygosity is not uncommon in AHS, which complicates the use of CRISPR-Cas-based approaches to fully recapitulate the disease’s genetic background.

## Reprogramming of patient-derived cells

Reprogramming of patient-derived cells into neurons is the other approach to model POLG-SDs *in vitro*. Patient-derived cells carrying *POLG* mutations, commonly fibroblast cells, are sampled from patients and cultivated in cell culture conditions followed by reprogramming the cells into iPSCs and differentiating them into neuronal cells [7]. Due to the limited access to human tissues, especially neural tissue, and the relative convenience of the sampling procedure (skin punch biopsy) [24], fibroblast cells are commonly preferred. Again, patient-derived fibroblasts are reprogrammed into iPSCs as outlined above [14], [15]. As patient-derived iPSCs are usually created by a scientific laboratory or a core facility serving a laboratory with a specific mainly scientific question, these cells are not characterized by a community of scientists. Thus, these individually reprogrammed iPSC lines are mainly used by one or a couple of laboratories, and molecular and functional workup naturally is restricted to some standard characterization of iPSCs (like basic pluripotency and rudimentary screenings for off-target editing and genetic aberrations) and functional assays that would answer the scientific question of the scientist/laboratory that initiated the iPSC generation. As a standard for iPSC lines, reprogrammed cells should be well characterized to rule out reprogramming artifacts and genetic aberrations. For instance, pluripotency is tested with the specific pluripotency markers *POU5F1* and *SSEA4* [25]. Also, the newly established cells must be expanded from single cells [26] in order to minimize the variation from the (mainly retroviral-based) reprogramming and possibly also the patient tissue. After the successful generation and characterization of patient-derived iPSCs, these cells (similarly to CRISPR-edited iPSCs as outlined above) could be differentiated into disease-related neurons, e. g., dopaminergic (DA) and cortical neurons [4], [5], for the study of POLG-SDs. Recently, Bindoff et al. [7] reported that patient-derived reprogrammed and differentiated DA neurons of the substantia nigra displayed defects with both lower mitochondrial DNA copy number and lower TFAM levels than control DA neurons. Their findings showed energy failure with loss of ATP production in POLG-specific iPSCs, but surprisingly, no change in mitochondrial DNA level or mitochondrial volume was detected. These findings also suggest that the pluripotent stage is not suitable for modeling POLG-SDs. However, reprogramming and differentiation processes can be time consuming and require careful daily maintenance. On the other hand, a patient-derived cell model does not require any genome engineering and already carries disease causing *POLG* mutations, which may not only include homozygous, but also still difficult-to-engineer compound heterozygous mutations. This advantage, however, does not come without a cost: At the end scientists want to rule out that (especially subtle) phenotypes of the patient-derived neurons are really due to the (*POLG*) mutation, and not due to possible human-to-human variability. Thus, CRISPR-based gene editing is required to correct the mutation of the iPSC-line to be studied, in order to be able to do meaningful control experiments with perfect control iPSCs that are “isogenic,” except for the patient mutation.

The major advantage of iPSCs and derived cells is the human (tissue-specific) environment in a close to physiological state, which is especially true when comparing iPSC-derived cells to cell lines usually derived from cancer cells. Modeling of complex inter-tissue interactions, e. g., the interaction of different neuronal subtypes, astrocytes, oligodendrocytes, and vasculature, in a human-like fashion is currently not feasible. Major efforts are made to generate organoids or chamber-based cell culture systems that would allow studying the interaction of two or more cell or tissue types making use of iPSCs. Standard animal models like *C. elegans*, *D. melanogaster*, *M. musculus*, and larger animals have provided valuable insights into a variety of physiological and pathomechanistic processes, but do not enable the study of human genes and human disease. A possibility to study human genes in animals is to replace the endogenous gene with the human ortholog (knockout/knockin), which has been done in numerous studies. While fascinating and insightful studies are being conducted with these animals, one can never be sure that interaction of the human (trans)gene or its product with the animal tissue/environment is substantially different compared to human tissue. Now, human cells derived from human iPSCs with their potential to differentiate into any given cell type or tissue may constitute a first-in-line platform for the study and therapy of human disease, providing valuable, human-specific pathomechanistic insights.

In sum, two of the most common and promising modeling approaches for human cell models are covered in this perspective: (i) CRISPR-Cas-based and iPSC-derived neuronal models and (ii) reprogramming of patient-derived cells into neurons. The latter usually also requires gene editing to obtain isogenic control cells. Despite the fact that cell models are in vitro systems incapable of recapitulating all aspects of neurogenetic diseases, they are still the reasonable point of start to discover disease mechanisms and develop therapeutic approaches to treat neurogenetic diseases.
